# The Pivotal Role of Benzimidazole in Improving the Thermal and Dielectric Performance of Upilex-Type Polyimide

**DOI:** 10.3390/polym15102343

**Published:** 2023-05-17

**Authors:** Meng Lian, Fei Zhao, Jun Liu, Faqin Tong, Lingbin Meng, Yongqi Yang, Feng Zheng

**Affiliations:** 1 Shandong Engineering Laboratory for Clean Utilization of Chemical Resources, Weifang University of Science and Technology, Weifang 262700, China; menglian@alumni.sjtu.edu.cn (M.L.); zhaofei@wfust.edu.cn (F.Z.); junliu@smail.nju.edu.cn (J.L.); mlb8124@126.com (L.M.); yongqiyang@wfust.edu.cn (Y.Y.); 2 Shanghai Sinochem Technology Co., Ltd., Kangwei Road 299, Pudong New District, Shanghai 201210, China; lanfeng@tongji.edu.cn; 3 School of Chemical Science and Engineering, Tongji University, Siping Road 1239, Shanghai 200092, China

**Keywords:** upilex-type polyimide, benzimidazole-containing diamine, conjugation system, hydrogen bond

## Abstract

Polyimide (PI) with ultra-high thermal resistance and stability is essential for application as a flexible substrate in electronic devices. Here, the Upilex-type polyimides, which contained flexibly “twisted” 4,4′-oxydianiline (ODA), have achieved various performance improvements via copolymerization with a diamine containing benzimidazole structure. With the rigid benzimidazole-based diamine bearing conjugated heterocyclic moieties and hydrogen bond donors fused into the PI backbone, the benzimidazole-containing PI showed outstanding thermal, mechanical, and dielectric performance. Specifically, the PI containing 50% bis-benzimidazole diamine achieved a 5% decomposition temperature at 554 °C, an excellent high glass transition temperature of 448 °C, and a coefficient of thermal expansion lowered to 16.1 ppm/K. Meanwhile, the tensile strength and modulus of the PI films containing 50% mono-benzimidazole diamine increased to 148.6 MPa and 4.1 GPa, respectively. Due to the synergistic effect of rigid benzimidazole and hinged, flexible ODA, all PI films exhibited an elongation at break above 4.3%. The electrical insulation of the PI films was also improved with a dielectric constant lowered to 1.29. In summary, with appropriate mixing of rigid and flexible moieties in the PI backbone, all the PI films showed superior thermal stability, excellent flexibility, and acceptable electrical insulation.

## 1. Introduction

Flexible electronic devices raise many challenges for the development of new materials with superior properties. Currently, polymer films have replaced the rigid glass part of traditional electronics and become ideal substrate materials for flexible electronic devices. Among polymer substrates, polyimide (PI) has outstanding comprehensive properties, including high-temperature resistance, excellent mechanical strength and flexibility, high radiation and wear stability. Therefore, the PI substrate has been widely used in flexible bandpass filters of wireless communications [1,2], thin-film transistors of flexible organic light-emitting diodes (OLEDs) [3,4], solar cells [5] etc. [6,7]. Many challenges remain in the application of PI films as flexible substrates, in which it is more imperative to enhance their thermal stability for special applications. For example, in the field of OLEDs, it has been verified that low-temperature poly Si (LTPS TFT) on PI substrates with high mobility and stability is indispensable for high definition display applications [8]. However, the LTPS TFT backplanes have been processed at relatively high temperature (≥450 °C) [4]. In the field of copper indium gallium di-selenide (CIGS) solar cells, the substrate must withstand the high temperature (≥550 °C) environment during absorber fabrication [5].

Upilex-R polyimide, derived from 4,4′-oxydianiline (ODA) and 3,3′,4,4′-biphenyltetracarboxylic dianhydride (BPDA), usually exhibits superior chemical, physical, and mechanical properties owing to the combination of rigid and linear chain structures, high molecular orientation, and ordered chain packing [9]. However, these polyimides often have inherently poor thermal properties due to the flexible linkage structure in ODA. Virtually all the excellent thermal properties of PI films are derived from traditional Van der Waals forces and charge-transfer forces between molecules. Therefore, extensive efforts have been made to improve PI performance by manipulating the structure of the molecular chain, adopting micro/nanoparticles and optimizing the imidization process. Among them, strengthening the stiffness of the intrinsic polyimide chain by monomer modulation is regarded as the best way to improve thermal performance. Liu et al. reported a new rigid quinoxaline diamine, from which high thermal-resistant polyimides could be prepared. However, some high *T*_g_ polyimides were too fragile to undergo tensile tests [10]. Li et al. demonstrated that the thermal performance of porous PI films could be improved by incorporating highly rigid benzimidazole units into the PI structure [11]. In addition, it has been shown that the thermal resistance of PI films can be enhanced by using benzimidazole-based diamines, and the thermal expansion behavior can be adjusted by changing the annealing temperature [12]. Wang et al. synthesized a series of thermally stable PIs containing benzimidazole moieties, but some of these PIs were too brittle to form self-standing films due to their highly rigid structure [13]. To compensate for the rigidity of the heteroaromatic ring and obtain a high *T*_g_ PI, Sidra et al. introduced a flexibly twisted structure -O- into special diamines [14] and dianhydrides [15] of the PI chains. In practical terms however, it led to counterproductive results, i.e., the *T*_g_ of some polyimides was not improved, or even worsened, due to the flexible bridge unit. Apparently, the flexible structure is somewhat non-conducive to thermal performance but beneficial to mechanical properties. Therefore, it is effective to mix stiff and flexible structures appropriately to balance or coordinate various performance enhancements.

In this work, we mixed rigid rod-like and flexible linkage structures in PI backbones by altering Upilex-R polyimide with geometrically asymmetric 2-(4-aminophenyl)-5-aminobenzimidazole (PABZ) or symmetric bis-benzimidazole diamine 4,4′-[5,5′-bi-1H-benzimidazole]-2,2′-diylbis-benzenamine (BB). These new PI films combined the flexible ether linkage and the rigid heterocyclic benzimidazole ring, as well as interchain hydrogen bond interactions that offered a compromise between high mechanical performance and enhanced thermal properties, including high *T*_g_, thermal stability, and low CTE. Furthermore, the effect of benzimidazole moieties on water absorption and dielectric properties was also systematically investigated.

## 2. Materials and Characterization

### 2.1. Materials

2-(4-aminophenyl)-5-aminobenzimidazole (PABZ), 4,4′-oxydianiline (ODA) and 3,3′,4,4′-biphenyl tetracarboxylic dianhydride (BPDA) were obtained from ChinaTech Chemical Co., Ltd. (Tianjin, China). *N,N*-dimethylacetamide (DMAc) with a trace of water ≤50 ppm was provided by Adamas-beta^®^ (Shanghai, China). All the above materials were used directly without further purification. Synthesis and characterization of 4,4′-[5,5′-bi-1H-benzimidazole]-2,2′-diylbis-benzenamine (BB) was according to the literature [16].

### 2.2. Characterization

The structure of PI films were identified on a Nicolet 6700 FTIR spectrometer (Thermo Fisher, Waltham, MA, USA) in attenuated total reflectance (ATR) mode with 32 scans over the range from 4000 to 650 cm^−1^. The lightness index (L^*^) was obtained from an UltraScan Pro spectrophotometer (Hunterlab, Reston, VA, USA). Wide-angle X-ray diffraction (WAXD) was performed on X`Pert^3^ Powder (PANalytical, Almelo, Netherlands). The glass-transition temperature (*T*_g_) was estimated by dynamic mechanical analysis (DMA) in a Q800 (TA Instrument, New Castle, DE, USA) at a heating rate of 5 °C/min under a nitrogen stream. A Discovery 550 TGA (TA Instrument) was applied to investigate the decomposition behavior of PI films at a temperature increase of 10 °C/min under nitrogen. The film’s coefficient of thermal expansion (CTE) was collected on a Q400 TMA (TA Instrument) at a heating rate of 5 °C/min with a static load of 0.05 N and a nitrogen flow of 50 mL/min. Mechanical properties were collected on a universal electromechanical WDW-50 (YiNuo, Jinan, China) with six specimens to obtain average values. The dielectric measurements were implemented on an E4990A Impedance Analyzer (Keysight, Santa Rosa, CA, USA) using the parallel-plate capacitor method in a dry vacuum chamber at a frequency from 0.01 to 10^6^ Hz. Gold electrodes were vacuum-deposited on both surfaces of dried films. The water-absorption ratio was calculated by the percentage of mass change before and after treatment by immersing 50 mm × 60 mm specimens into a distilled water bath at 25 °C for 24 h.

### 2.3. Polyimide Preparation

**Figure 1:**
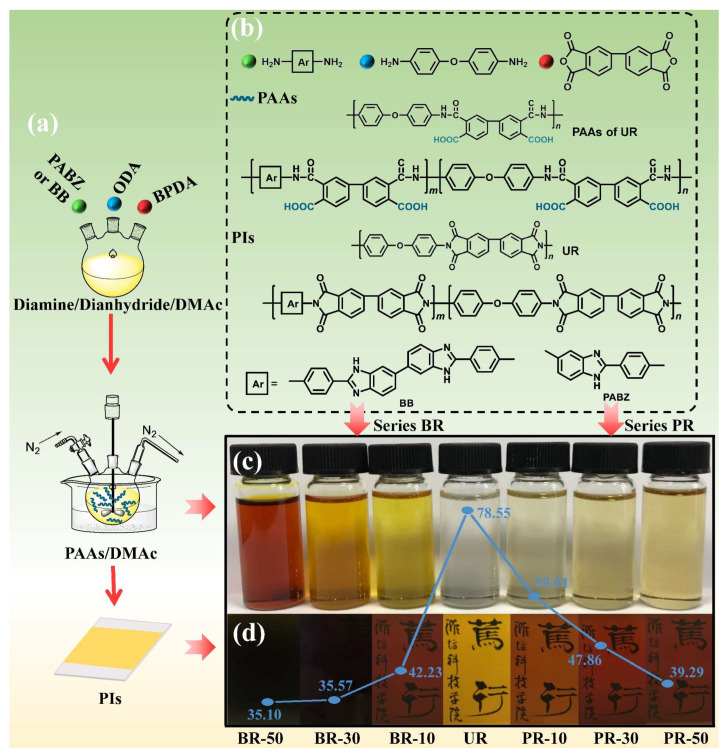
Polyimide fabrication. (**a**) PI films obtained by a two-step method: polymerization to obtain PAAs initially and subsequent imidization to obtain PI films. (**b**) Chemical structures of diamine, dianhydride, PAAs, and PI films. Visible images of PAAs (**c**), corresponding PI films (**d**), and lightness index of PI films are shown in blue. The Chinese characters in (**d**) represents the Chinese name of “Weifang University of Science and Technology”, which were seen through the PI films and used for visual comparison of the darkness of PI films.

Polyimides were synthesized through a traditional two-step method, as shown in Figure 1a. Diamine ODA and benzimidazole-containing diamine (PABZ or BB) were mixed and stirred with equimolar amounts of dianhydride BPDA in anhydrous DMAc to prepare poly(amic acid)s (PAAs). The solid content of the PAAs ranged from 8wt% to 13wt% and was then homogeneously cast onto the glass plate. The PAAs were dried in an established preheating program and a progressive thermal treatment was applied to these layers in a muffle. The structures of diamine, dianhydride, PAAs and polyimides are shown in Figure 1b. According to the structure of the diamine copolymerized with ODA, all copolyimides were divided into two series, i.e., the PR series polyimides with mono-benzimidazole and the BR series polyimides with bis-benzimidazole. These copolyimides were named according to the percentage of diamine containing benzimidazole, and the molar ratios with ODA were m:n (m:n = 1:10, 3:10, and 5:10). The homopolyimide, that is, m = 0, was named UR as a control sample, which maintains the same structure as the Upilex-R polyimide.

## 3. Results and Discussion

### 3.1. Polyimide Characterization

From the photographs of PAAs and their corresponding PI films shown in Figure 1c,d, it can be seen that the color of PAAs and PI films became darker as the content of benzo-fused diamine PABZ and BB increased. In addition, the lightness index (L^*^) also reflects the perception of the light source. A darker color leads to a lower lightness index and vice versa. As shown in Figure 1d, the L^*^ of polyimides, in line with the gradually deepened color displayed, decreased from 78.55 to 39.29 as the PABZ content increased and to 35.10 as the BB content increased. Generally, the larger the conjugation system, the higher the extinction coefficient [17,18], and apparently the darker coloration. Therefore, the diamine containing benzimidazoles offer high conjugation to polymer chains, and the conjugated heterocyclics of the BR series were much higher than the PR series.

**Figure 2:**
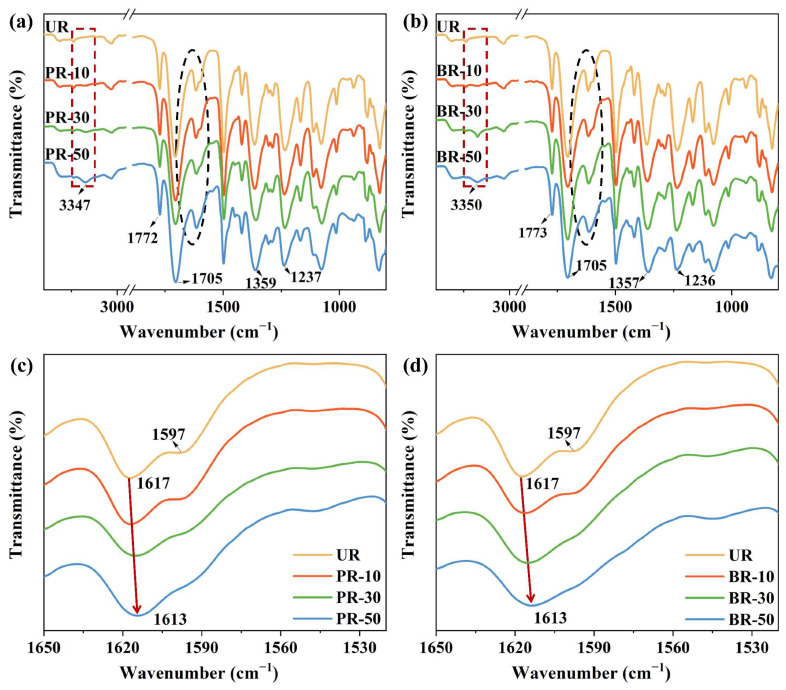
ATR-FTIR spectra of PI films: (**a**) PR series and (**b**) BR series. The transmission spectra (elliptical area) of PI films in the wavenumber range from 1650 cm^−1^ to 1530 cm^−1^: (**c**) PR series and (**d**) BR series (the vibrational changes of benzene skeleton were indicated by red arrows).

The ATR-FTIR spectra also illustrated the enhanced conjugation system of PR series and BR series polyimides. First, as shown in Figure 2, all PI films exhibited characteristic peaks of the imide ring at 1359 cm^−1^ (C–N axial stretching), 1705 cm^−1^ and 1772 cm^−1^ (C=O stretching) [19,20]. The peaks at 1237 cm^−1^ (C–O–C stretching) and 3347 cm^−1^ (N–H stretching) indicate the presence of ODA and benzimidazole, respectively [21]. Moreover, with the increase in the content of PABZ or BB, the band of N–H in benzimidazole became stronger, and a broad stretching band was observed. This illustrated the successful incorporation of benzimidazole segments into the main chain of the polyimide and the formation of hydrogen bonds between the N atom of benzimidazole moieties and the O atom of imide rings (N–H
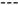
O=C). Last but not least, the peak corresponding to the benzene skeleton vibration at 1617 cm^−1^ and 1597 cm^−1^, shifted to 1613 cm^−1^ and nearly disappeared, respectively, with the increase in benzimidazole moieties in both series (from UR to PR-50 and to BR-50). According to the literature [22], conjugation usually leads to a decrease in the chemical bond force constant, and the IR peak shifts to a lower frequency. Therefore, the results also confirm that the conjugation system is enlarged after copolymerization with benzimidazole-bearing diamine.

**Figure 3:**
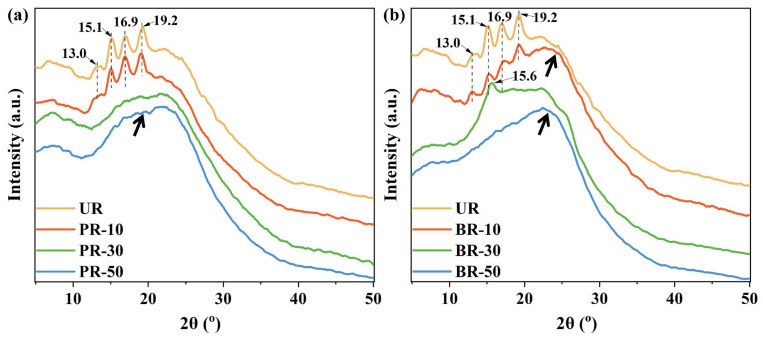
WAXD profiles of PI films: (**a**) PR series and (**b**) BR series (the black arrows indicate the maximum of the amorphous broadband peak).

To probe the molecular packing, wide-angle X-ray diffraction (WAXD) of the polyimides was measured and is shown in Figure 3. First, the homopolyimide UR for reference exhibited four clear diffraction peaks at approximately 13.0° (6.8 Å), 15.1° (5.9 Å), 16.9° (5.2 Å), 19.2° (4.6 Å). These peaks were in good agreement with the “side-to-side” distance of aromatic polyimides [23,24], indicating that some well-defined lateral long-range order molecular packing was present in the UR polyimide. After copolymerization with PABZ or BB, these peaks became weaker (e.g., BR-10), partially disappeared (e.g., PR-10 and BR-30) and completely disappeared (e.g., PR-30, PR-50, and BR-50). It implies that the highly ordered structure was disturbed with the benzimidazole moieties incorporated into the UR backbone, resulting in the decrease of crystallinity, and the polyimides consequently becoming amorphous. This agrees well with the previous report that the aromatic and bulky segments can cause Upilex-type films to be amorphous [25]. Furthermore, the BR series polyimides were expected to have a more condensed molecular packing compared to the PR series polyimides, as the BR series would have more hydrogen bonds and enlarged conjugation arising from the bis-benzimidazole in diamine BB. This speculation can be proven by the *d*-spacing obtained in WAXD, which reflects the average intermolecular distance and is indicative of the free volume [26,27]. Since the maximum of the amorphous broadband peak in the BR series was higher than that in the PR series, the BR series polyimides exhibit a lower *d*-spacing. The sharp peak of BR-30 was at 15.6° (the *d*-spacing was 5.7 Å), which was also higher than 15.1° (the *d*-spacing was 5.9 Å) for UR, PR-10. Thus, the packing density of the BR series was higher than that of the PR series as the stronger interchain interactions from hydrogen bonds and the dilated conjugation system can effectively enhance polymer chain packing.

### 3.2. Thermal Properties

**Figure 4:**
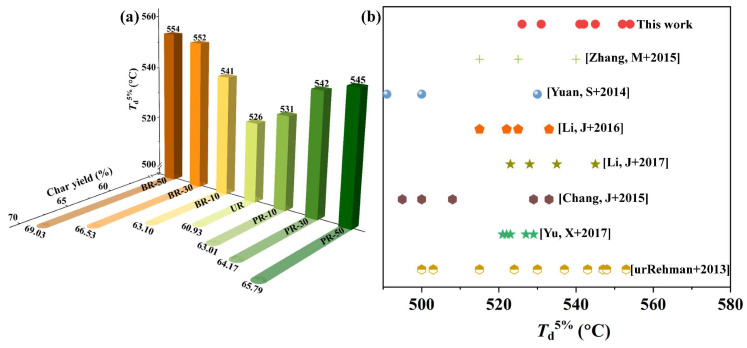
The thermal decomposition of PI films. (**a**) 5% decomposition temperature (column) and char yield when heated to 800 °C (column shadow) of polyimides. (**b**) 5% decomposition temperature contrast between our work and other published works [11,28,29,30,31,32,33] on polyimides containing benzimidazole moieties and ODA.

First, the effects of benzimidazole segments on the thermal decomposition of PI films was investigated. The results in Figure 4a and Figure S1 (see in Supplementary Materials), show the 5% weight loss temperature (*T*_d_^5%^) of the PI films improved by different degrees compared to the UR PI after incorporating benzimidazole segments to the polymer chains. In particular, the *T*_d_^5%^ values of BR-50 and PR-50 films are as high as 554 °C and 545 °C, respectively, which rank at a relatively high level among polyimides containing benzimidazole moieties and ODA, as shown in Figure 4b [11,28,29,30,31,32,33]. Such high *T*_d_^5%^ values can be accounted for by the benzo-fused heterocyclic ring without hinging atoms, which exhibits an activation barrier to decomposition due to the C–N and C=N in imidazole possessing shorter bond lengths and higher bond energies compared with C–O and C=C in ODA [10,34,35]. In addition, as described in the color and ATR-FTIR spectrum section, the benzimidazole group provided a higher conjugation system, which made the thermal energy distribution to other bonds easier and prevented polymer chains from thermal fractures [36,37]. At the same time, hydrogen bonds occurring at the N–H bond on the imidazole ring could effectively make molecular chains pack closer and hinder them dissociating. The thermal stability of PI films was improved after copolymerization with benzimidazole-containing diamine. The more conjugated and rigid aromatic benzimidazole rings in PI chains, the more hydrogen bonds and conjugation systems were formed. Therefore, the benzimidazole segments elevate the *T*_d_^5%^ values and residue weights of their corresponding PI films incrementally with the increasing PABZ/BB moieties. Another significant finding was that the *T*_d_^5%^ values and residue weight when heated to 800 °C of the BR series were higher than those of the PR series. Although it has been proven that the N–H group in the imidazole ring is more vulnerable to heat attack [38], and the content of carbon in ODA is slightly higher than that in PABZ and BB, the close packing of the polymer chains through hydrogen bond and interchain conjugation could effectively prevent chain-scission degradation. Therefore, some heteroatoms in the polymer chain remained, leading to a higher residue weight in the BR series, which has a larger conjugation system and more hydrogen bonds than the PR series.

**Figure 5:**
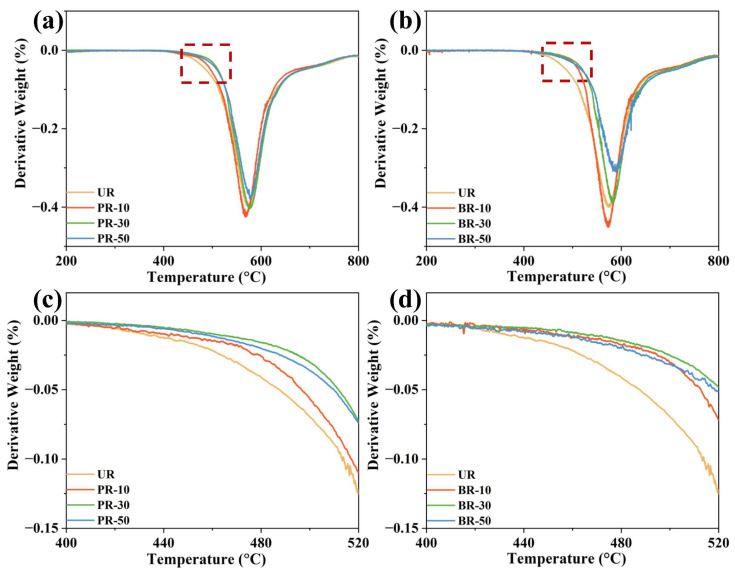
The derivative thermal gravity analysis (DTG) of PI films: (**a**) PR series and (**b**) BR series. The DTG curves in the temperature range from 400 °C to 520 °C (rectangular area): (**c**) PR series and (**d**) BR series.

The derivative thermal gravity analysis (DTG) was performed to further investigate the thermal decomposition behavior, as shown in Figure 5. The major decomposition temperature (*T*_max_) of UR was observed at 574 °C, while PR-10, PR-30, PR-50, BR-10, BR-30 and BR-50 exhibited *T*_max_ of 572 °C, 577 °C, 580 °C, 574 °C, 583 °C and 589 °C, respectively. The significant weight loss was due to the disintegration of aromatic rings and molecular chains. Although the shape of all DTG curves was similar, it can be observed that the weight loss rate differed greatly. In the initial period of weight loss, the hydrogen abstraction of N–H groups [38] and the decomposition of “pin-joint” –O– bonds [36] were the main cause of weight loss. As shown in Figure 5c,d, the weight loss rate was arranged in the sequence of UR > PR-10 > PR-50 > PR-30 for the PR series in the temperature range of 400 °C to 520 °C. For the BR series, the weight loss rate decreased in the order of UR > PR-50 > PR-10 > PR-30 from 400 °C to 500 °C, while it was UR > PR-10 > PR-50 > PR-30 for the temperature range of 500°C to 520 °C. It can be inferred that the temperature of –O– decomposition is lower than that of imidazolic N–H. Moreover, the stability of N–H groups can also be enhanced by the formation of hydrogen bonds. Therefore, the *T*_d_^5^% and *T*_max_ improved after the incorporation of benzimidazole moieties. However, the formation of hydrogen bonds was susceptible to intermolecular distance and bond angles [39]. Too few benzimidazole rings could lead to a higher degree of free N–H, which could cause a decrease in *T*_max_. Therefore, the *T*_max_ of PR-10 was slightly lower than that of UR, and the weight loss rate of PR-10 and BR-10 was higher in their corresponding series. The more benzimidazole rings, the more radical species formed and evolved into more stable quinone and benzonitrile structures when heated [38], leading to a higher weight loss rate at first and lower later, as observed in PR-50 and BR-50. For PR-30, the weight loss rate was the slowest in the early period and then experienced a high decomposition at temperatures higher than 530 °C. Therefore, a slightly improved degree of *T*_d_^5%^ was exhibited from PR/BR-30 to PR/BR-50.

One of the most important attributes to measure the thermal resistance of new PI films is their glass temperature (*T*_g_), which reflects the upper operational temperature for amorphous polymers. High thermal resistance (i.e., high *T*_g_) is an indispensable requirement for PI substrates to avoid distortion as little as possible during the sequential high-temperature processing steps of flexible OLEDs and solar cells. Dynamic mechanical analysis (DMA) was conducted on these PI films, and the α-relaxation (main peak) values of tan δ were assigned as *T*_g_ values (Figure S2, see in Supplementary Materials). In DMA analysis, changes in tan δ are caused by either a relaxation transition (e.g., a glass transition) or a phase transition (*e.g*., melting and crystallization). In general, several relaxation transitions can be observed for amorphous and semi-crystalline materials. Usually, the obvious relaxation transition at the highest temperature is generally referred to as the α-transition or glass transition, which is caused by the coordinated motion of molecules on the supernanometric scale. The weaker secondary relaxation, called β-relaxation, is caused by the movement of side groups, short segments, etc. As shown in Figure S2 (see in Supplementary Materials), two peaks of the tan δ curve can be observed obviously in UR and BR-10, with the latter being weaker. Meanwhile, considering that the properties of the PI films have changed dramatically and lost operating capability when the temperature rises to the formation of the first peak, the first peak was taken as the *T*_g_ value of UR and BR-10. The two peaks suggest that there are two phases in UR. As shown in XRD analysis, the two phases gradually changed to be amorphous with the introduction of PABZ or BB. For PR-10 and PR-30, several repeated measurements showed that after the first peak of the tan δ curve appeared, the sample in DMA Q800 was stretched to fracture when the maximum measured temperature was not reached at 500 °C and loss signals (the curve breaks into a straight line). It is reasonable to infer from the existing curve trends that a second peak would appear near 500 °C and the two phases existed when the content of PABZ or BB was lower, such as BR-10, PR-10, and PR-30. Meanwhile, the tan δ curves of PR-50 and BR-50 exhibit a perfect single peak.

**Figure 6:**
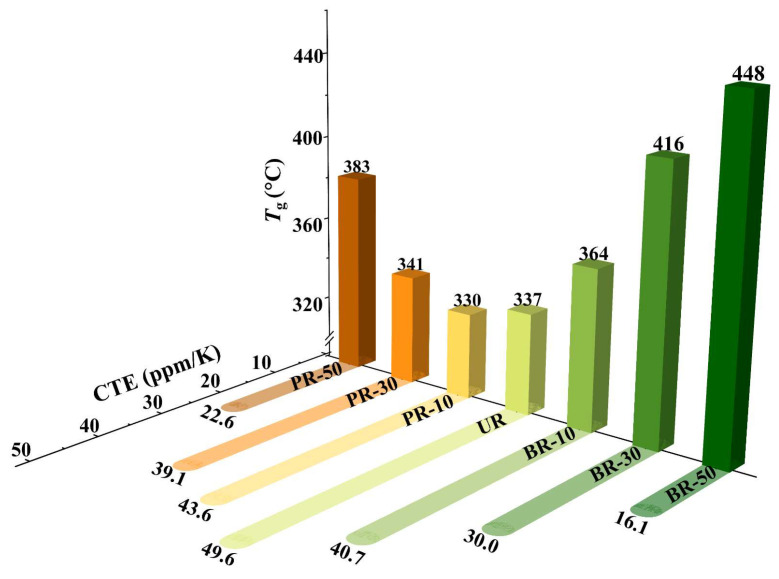
*T*_g_ and CTE of polyimides.

As summarized in Figure 6, most polyimides containing benzimidazole exhibited high *T*_g_ compared to PIs with flexible ether linkages [29,40]. The *T*_g_ increased monotonically from 337 °C to 448 °C for the BR series. However, for the PR series, *T*_g_ decreased slightly from 337 °C to 330 °C for PR-10, and then increased to 383 °C when the PABZ content reached 50%. On one hand, the heterocycle rigidity, conjugation, and intramolecular electron donation–acceptance interaction (i.e., hydrogen bonding) was enhanced after incorporating the tectonic benzimidazole unit. The strengthened inter/intramolecular interactions restrict polymer chain-segment motion and increase *T*_g_ accordingly. On the other hand, as shown in the XRD results, PABZ or BB moieties disturbed the organized homopolymer chains and decreased the crystallization, leading to a decrease in *T*_g_. These two factors compete and coordinate with each other, leading to *T*_g_ increasing by approximately 111 °C for the BR series and decreasing slightly for PR-10 but increasing by 4 °C and 46 °C for PR-30 and PR-50, respectively.

Polyimide substrates are commonly laminated or evaporated with other materials for further thermal processing. To reduce peeling and warpage derived from the smaller dimensional changes of inorganic or metal layers, the coefficient of thermal expansion (CTE) is another key parameter for measuring the thermal performance of PI films. The CTE values were calculated from the results of thermomechanical analysis (TMA) in Figure S3 (see in Supplementary Materials) in the range from 50 °C to 350 °C, as shown in Figure 6. It is well known that the CTE value depends on the variation of the polymer chain in-plane orientation, which is related to interchain interactions and chain linearity/stiffness [41,42,43]. In this case, due to the flexible and sterically twisted structure of ODA, the homopolyimide UR displayed the highest CTE values (49.6 ppm/K). Furthermore, due to the linear and rigid structure of the benzimidazole ring, as well as the strengthening effect of hydrogen bonds on polymer interaction, the copolyimides containing heterocyclic segments have the potential to significantly enhance the spontaneous molecular chain orientation during thermal imidization. As a result, they effectively suppress the thermal expansion of the polyimide matrix. Therefore, the CTE values of the PR and BR series PI films decreased greatly with an increase in benzimidazole content. For example, when the benzimidazole content reached 50%, the CTE dropped to 22.6 ppm/K for PR-50, and to as low as 16.1 ppm/K for BR-50. In addition, the CTE of the BR series was perpetually lower than that of the PR series under the same content of PABZ/BB, as the higher tectonic linearity of bis-benzimidazole with more hydrogen bonds in the BR series than mono-benzimidazole in the PR series is beneficial for chain in-plane orientation and resulted in lower CTE.

### 3.3. Mechanical Properties

**Figure 7:**
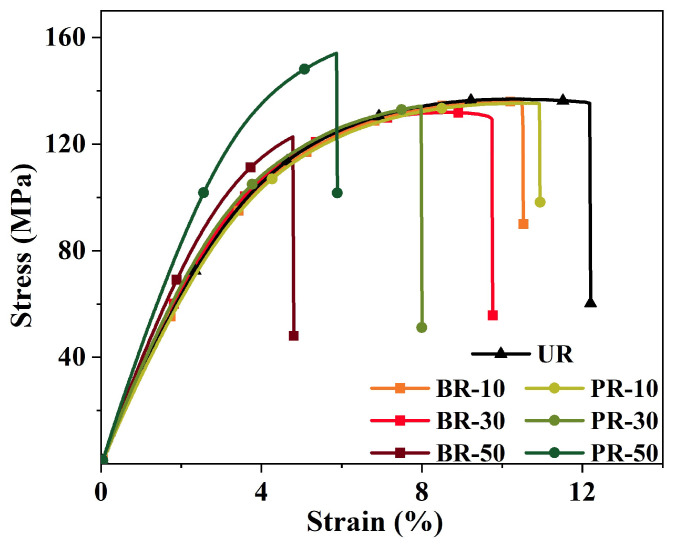
Tensile strain-stress curves of one sample in six PI film specimens.

The mechanical properties were also a hard index for practical application of PI films in the advanced manufacturing industry. The tensile stress–strain curves are shown in Figure 7. The corresponding tensile properties are tabulated in Table 1. All the prepared PI films display superior mechanical properties with tensile strengths ranging from 118.9 MPa to 148.6 MPa and a tensile modulus from 2.7 GPa to 4.1 GPa. Factors such as enhanced intermolecular interactions, polymer chain stiffness, and high molecular weight are beneficial for improving the mechanical properties of polyimides, but a PI film with a high-level rigid structure is always delicate. Here, all the PI films still have excellent mechanical performance and adequate flexibility. The elongation at the break of PI films was greater than 4.3% despite displaying a decreasing trend with an increase in benzimidazole segment loading. Moreover, the existence of a rigid benzimidazole induces strong hydrogen bond interactions within the polymer system, which act as physical cross-links and cause polymer chain entanglements [15,23]. Thereby, the tensile strength of PR series polyimides exhibited an obvious upward trend with the increasing benzimidazolyl diamine content. Meanwhile, the tensile strength of the BR series polyimides showed a slight decrease as a result of higher rigidity and higher number of hydrogen bond interactions of bis-benzimidazole moieties. In summary, rigid, conjugated benzimidazole moieties and dianhydride rings make PI chains highly stiff and guarantee the strength and modulus, while the flexible ODA structures make PI chains intertwine with each other, ensuring good flexibility.

### 3.4. Dielectric Properties

**Figure 8:**
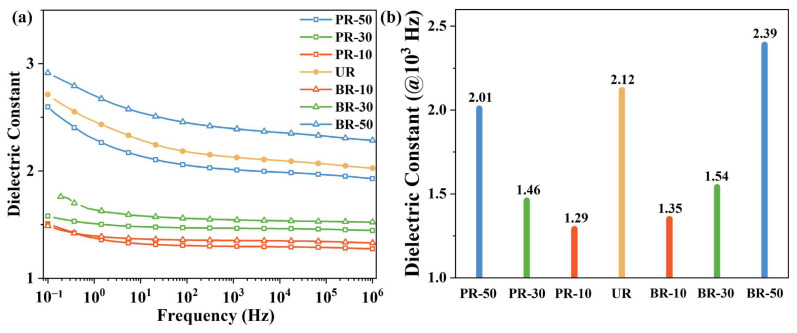
(**a**) Frequency scan broadband dielectric constant spectroscopy of polyimides and (**b**) dielectric constant at 10^3^ Hz.

The dielectric properties were also applied to evaluate the influence of benzimidazole moieties on electronic properties. The variation curves of the dielectric constant and dielectric loss of the PI films are shown in Figure 8a and Figure S4 (see in Supplementary Materials). The dielectric constant values of the PI films decreased with the increasing frequency attributed to the frequency dependence of polarization mechanism. That is, fewer dipole orientations in PI films can keep pace with the oscillation of alternating electric fields with the increasing frequency, which can reduce the function of orientational polarization and lead to dielectric constant decline [44]. Moreover, a longer repeating unit has shown a stronger ability to dilute the dipole density from carbonyl and imide structures [45]. As a consequence, the PI with a higher benzimidazole-containing diamine content exhibited a higher dielectric constant decline when the frequency increased from 0.01 to 10^6^ Hz.

The dielectric constant and dielectric loss values at 10^3^ Hz are listed in Figure 8b and Table 1. First, it has been noted that conjugation could elevate the dielectric constant because the conjugated structure has the largest permanent dipoles and is able to delocalize electrons [44]. Second, many examples have proven that reducing interchain interactions can effectively reduce dielectric constant by introducing flexible linkages [45,46], fluorinated structures [47,48], and bulky structures [48] in the polymer backbones. As a result, the dielectric constant of the PR series increased from 1.29 to 2.01, and that of the BR series from 1.35 to 2.39 due to the introduction of conjugated benzimidazole moieties and enhanced polymer chain interactions. As a result of bis-benzimidazole with a larger conjugation system, the dielectric constant values of the PR series were lower than those of the BR series with the same content of benzimidazolyl diamine. Finally, the UR film without conjugated benzimidazole exhibited a relatively high dielectric constant (2.12), which can be attributed to the partially regular-oriented polymer chains as evidenced by XRD analysis. The regular-oriented chains arise from the strong interchain interactions [45,48] which could increase the dielectric constant. However, when copolymerized with PABZ/BB, the well-organized structure in UR films was disrupted, resulting in PR-10 and BR-10 films showing a lower dielectric constant after the introduction of conjugated benzimidazole moieties.

### 3.5. Water Absorption Behavior

**Figure 9:**
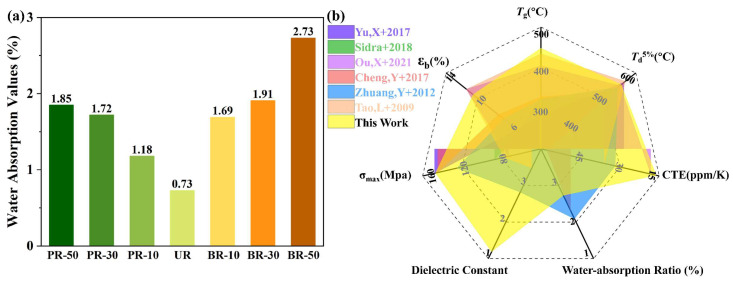
(**a**) Water absorption of polyimides; (**b**) schematic for the comparison of the properties of this work with reported results [15,20,29,40,52,53].

Apart from the thermal, mechanical and electronic properties of the polymer substrate films, their water absorption characteristics can affect the performance of electronic devices also, and thus was further investigated. As shown in Figure 9a, the water absorption value of all PI films ranged from 0.73 to 2.73, similar to most reported polyimides [32,49,50]. The water absorption behavior is susceptible not only to the polymer chain packing, but also to the concentration of polar moieties and hydrophilic units [51]. On the one hand, the homopolyimide UR, exhibits a highly ordered structure, which is more hydrophobic. On the other hand, the water affinity promoting N–H units in benzimidazole moieties tend to form hydrogen bonds with water molecules, giving rise to greater water absorption values. Consequently, the water-absorption ratios of PI films increased after incorporating benzimidazole-containing diamines. Notably, hydrogen bonding and conjugation have superior effects on water absorption compared to packing density. It was observed that polyimides in the BR series with a higher benzimidazole content still exhibit a higher water absorption ratio. Although these PI films have more hydrogen bonds and enlarged conjugations they show higher packing density than those of the PR series.

In fact, numerous methods have been employed to improve the thermal, mechanical, and even dielectric properties of PI films containing flexible, hinged ODA, such as the adoption of special monomers, introduction of nanocomposites and optimization of thermal or chemical imidization technology. Figure 9b displays the comparison of the thermal index (*T*_g_, *T*_d_^5%^, CTE), mechanical properties (tensile strength and elongation at break), water-absorption ratio and dielectric constant of films with reported results [15,20,29,40,52,53]. It is shown that the PI films containing BB have comparable thermal properties. The *T*_g_ reaches 448 °C and CTE reaches 16 ppm/K, which exceeds the value reported in most of the literature. Furthermore, it is important to note that while the elongation at break may not be exceptional, it is sufficient for certain applications, such as flexible OLED substrates that require a tensile strength >100 MPa, an initial modulus >2 GPa and elongation at break > 4%. In summary, the excellent thermal and dielectric properties demonstrate the beneficial effect of benzimidazole moieties on the performance of PI films. However, the impact of benzimidazole moieties on the mechanical properties of the films is influenced by the content and construction of the corresponding diamine. Therefore, to further research and investigate applications, an appropriate design of monomer ratio and structure are necessary.

## 4. Conclusions

In this work, the influence of benzimidazole segments on Upilex-type polyimides was systematically investigated, in which two series of PI films containing PABZ and BB were prepared. With the content of PABA/BB increased, the color of the PI films changed from yellow to brown, and the benzene skeleton vibration exhibited a redshift, indicating a larger conjugation system after incorporation of rigid rod-like benzimidazole segments. The WAXD peak gradually decreased or even disappeared, suggesting that the hydrogen bonds were formed between polyimide chains. With the hydrogen bond and rigid conjugated heterocyclic backbone, the *T*_d_^5%^ value of these PI films increased from 526 °C to 554 °C and *T*_g_ from 337 °C to 448 °C, while CTE decreased from 49.6 ppm/K to 16.1 ppm/K. All PI films show excellent mechanical performance and adequate flexibility to meet application requirements due to the synergistic effect of rigid benzimidazole with flexible hinged ODA. The tensile strength, modulus and elongation at break of all PI films were higher than 118.9 MPa, 2.7 GPa, and 4.3%, respectively. Furthermore, the dielectric properties also improved because benzimidazole disrupted the Upilex-R organized structure. Thus, benzimidazole-containing PI films exhibited a good combination of properties and demonstrated that mixing rigid and flexible structures appropriately can realize the targeted adjustment of performance.

## Figures and Tables

**Table 1:** Mechanical and dielectric properties of polyimide films.

PIs	σ_max_^a^ (MPa)	E ^b^ (GPa)	ε_b_^c^ (%)	Dielectric Loss (×10^−3^)
BR-50	118.9 ± 9.8	2.7 ± 0.6	4.3 ± 0.7	13.2
BR-30	128.1 ± 8.2	3.1 ± 0.4	6.0 ± 1.3	8.2
BR-10	136.0 ± 9.5	2.9 ± 0.6	10.4 ± 2.5	2.8
UR	132.9 ± 9.5	3.0 ± 0.5	11.7 ± 1.8	12.9
PR-10	133.4 ± 8.6	3.1 ± 0.3	10.8 ± 1.2	4.1
PR-30	137.7 ± 10.7	3.4 ± 0.5	9.3 ± 0.9	1.7
PR-50	148.6 ± 7.8	4.1 ± 0.7	6.2 ± 0.7	10.3

^a^ Tensile strength; ^b^ initial modulus; ^c^ elongation at break.

## Data Availability

The data presented in this study are available in this article and supporting information.

## Supplementary Materials

The following supporting information can be downloaded at: https://www.mdpi.com/article/10.3390/polym15102343/s1, The synthesis and characterization of 4,4′-[5,5′-bi-1H-benzimidazole]-2,2′-diylbis-benzenamine (BB); Figure S1: TGA curves of PI films (a) PR series and (b) BR series.; Figure S2: DMA curves of PI films (a) PR series and (b) BR series.; Figure S3: TMA curves of PI films (a) PR series and (b) BR series.; Figure S4: Frequency-scan broadband dielectric loss spectroscopy of polyimides.

## References

[1] Gong J.S., Wang Z.L., Qu H.W., Cang D.Y. (2021). Flexible bandpass filter on polyimide substrate. J. Mater. Sci. Mater. Electron..

[2] Gong J., Wang Z., Qu H., Cang D. (2021). The flexible bandpass filter on polyimide substrate for wireless communications systems. Semicond. Sci. Technol..

[3] Zou J., Zhang M., Zhao K., Zhang Q., Deng M., Huang F., Kang L., Hu Z., Zhang J., Li W. (2021). Flexible organic thin-film transistors with high mechanical stability on polyimide substrate by chemically plated silver electrodes. IEEE Trans. Electron Devices.

[4] Lee S., Cho Y.J., Han B., Lee J., Choi S., Kang T., Chu H.Y., Kwag J., Kim S.C., Jang J. (2021). Poly-Si thin-film transistors on polyimide substrate for 1 mm diameter rollable active-matrix organic light-emitting diode display. Adv. Eng. Mater..

[5] Shi S., Yao L., Ma P., Jiao Y., Zheng X., Ning D., Chen M., Sui F., Liu H., Yang C. (2021). Recent progress in the high-temperature-resistant PI substrate with low CTE for CIGS thin-film solar cells. Mater. Today Energy.

[6] Kubo Y., Sonohara Y., Uemura S. (2021). Changes in the chemical state of metallic Cr during deposition on a polyimide substrate: Full soft XPS and ToF-SIMS depth profiles. Appl. Surf. Sci..

[7] Yao Y., Guo W., Zhou X., Peng P. (2021). Thermal properties of laser-fabricated copper-arbon composite films on polyimide substrate. Adv. Eng. Mater..

[8] Powell M.J. (1989). The physics of amorphous-silicon thin-film transistors. IEEE Trans. Electron Devices.

[9] Dine-Hart R.A., Wright W.W. (1967). Preparation and fabrication of aromatic polyimides. J. Appl. Polym. Sci..

[10] Liu T.Q., Zheng F., Ding T.M., Zhang S.Y., Lu Q. (2019). Design and synthesis of a novel quinoxaline diamine and its polyimides with high-*T*_g_ and red color. Polymer.

[11] Li J., Zhang G., Jing Z., Li J., Zhou L., Zhang H. (2017). Synthesis and characterization of porous polyimide films containing benzimidazole moieties. High Perform. Polym..

[12] Song G., Zhang X., Wang D., Zhao X., Zhou H., Chen C., Dang G. (2014). Negative in-plane CTE of benzimidazole-based polyimide film and its thermal expansion behavior. Polymer.

[13] Wang S., Zhou H., Dang G., Chen C. (2009). Synthesis and characterization of thermally stable, high-modulus polyimides containing benzimidazole moieties. J. Polym. Sci. Part A Polym. Chem..

[14] Sidra L.R., Chen G., Li C., Mushtaq N., Ma K., Fang X. (2018). Processable, high *T*_g_ polyimides from unsymmetrical diamines containing 4-phenoxy aniline and benzimidazole moieties. Polymer.

[15] Sidra L.R., Chen G., Mushtaq N., Xu L., Chen X., Li Y., Fang X. (2018). High *T*_g_, melt processable copolyimides based on isomeric 3,3’and 4,4’-hydroquinone diphthalic anhydride (HQDPA). Polymer.

[16] Lian M., Zheng F., Lu X., Lu Q. (2019). Tuning the heat resistance properties of polyimides by intermolecular interaction strengthening for flexible substrate application. Polymer.

[17] Yue H., Kong L., Wang B., Yuan Q., Zhang Y., Du H., Dong Y., Zhao J. (2019). Synthesis and characterization of novel D-A type neutral blue electrochromic polymers containing pyrrole[3-c]pyrrole-1,4-diketone as the acceptor units and the aromatics donor units with different planar structures. Polymers.

[18] Hong K., Yu H.K., Lee I., Kim S., Kim Y., Kim K., Lee J.L. (2018). Flexible top-emitting organic light emitting diodes with a functional dielectric reflector on a metal foil substrate. RSC Adv..

[19] Chen H., Dai F., Yan X., Chen C., Qian G., Yu Y. (2021). Novel semi-N-methyl substituted bisbenzimidazole based polyimide films with low coefficient of thermal expansion and high *T*_g_. J. Polym. Res..

[20] Tao L., Yang H., Liu J., Fan L., Yang S. (2009). Synthesis and characterization of highly optical transparent and low dielectric constant fluorinated polyimides. Polymer.

[21] Zhou Y., Zhang S., Zheng F., Lu Q. (2021). Intrinsically black polyimide with retained insulation and thermal properties: A black anthraquinone derivative capable of linear copolymerization. Macromolecules.

[22] Snels M., Beil A., Hollenstein H., Quack M. (1997). Excited vibrational states of benzene: High resolution FTIR spectra and analysis of some out-of-plane vibrational fundamentals of C_6_H_5_D. Chem. Phys..

[23] Feng Y., Luo L.B., Huang J., Li K., Li B., Wang H., Liu X. (2016). Effect of molecular rigidity and hydrogen bond interaction on mechanical properties of polyimide fibers. J. Appl. Polym. Sci..

[24] Wakita J., Jin S., Shin T.J., Ree M., Ando S. (2010). Analysis of molecular aggregation structures of fully aromatic and aemialiphatic polyimide films with synchrotron grazing incidence wide-angle X-ray scattering. Macromolecules.

[25] Feng J., Wang Y., Qin X., Lv Y., Huang Y., Yang Q., Li G., Kong M. (2022). Property evolution and molecular mechanisms of aluminized colorless transparent polyimide under space ultraviolet irradiation. Polym. Degrad. Stab..

[26] Chen Y., Zhang Q., Sun W., Lei X., Yao P. (2014). Synthesis and gas permeation properties of hyperbranched polyimides membranes from a novel (A_2_+B_2_B’+B_2_)-type method. J. Membr. Sci..

[27] Lei X., Qiao M., Tian L., Chen Y., Zhang Q. (2016). Tunable permittivity in high-performance hyperbranched polyimide films by adjusting backbone rigidity. J. Phys. Chem. C.

[28] Zhang M., Niu H., Chang J., Ge Q., Cao L., Wu D. (2015). High-performance fibers based on copolyimides containing benzimidazole and ether moieties: Molecular packing, morphology, hydrogen-bonding interactions and properties. Polym. Eng. Sci..

[29] Yu X., Liang W., Cao J., Wu D. (2017). Mixed rigid and flexible component design for high-performance polyimide films. Polymers.

[30] Yuan S., Guo X., Aili D., Pan C., Li Q., Fang J. (2014). Poly(imide benzimidazole)s for high temperature polymer electrolyte membrane fuel cells. J. Membr. Sci..

[31] Chang J., Liu W., Zhang M., Cao L., Ge Q., Niu H., Sui G., Wu D. (2015). Structures and properties of polyimide fibers containing fluorine groups. RSC Adv..

[32] UrRehman S., Song G., Jia H., Zhou H., Zhao X., Dang G., Chen C. (2013). Synthesis and characterization of benzimidazole-based low CTE block copolyimides. J. Appl. Polym. Sci..

[33] Li J., Zhang G., Yao Y., Jing Z., Zhou L., Ma Z. (2016). Synthesis and properties of polyimide foams containing benzimidazole units. RSC Adv..

[34] Slonimskii G.L., Askadskii A.A., Kitaigorodskii A.I. (1970). The packing of polymer molecules. Polym. Sci. U.S.S.R..

[35] Bondi A. (1964). Van der Waals volumes and radii. J. Phys. Chem..

[36] Sazanov Y.N., Florinsky F.S., Koton M.M. (1979). Investigation of thermal and thermooxidative degradation of some polyimides containing oxyphenylene groups in the main chain. Eur. Polym. J..

[37] Varma I.K., Goel R.N., Varma D.S. (1979). Effect of structure on the thermal stability of polyimides. J. Polym. Sci. Polym. Chem. Ed..

[38] Musto P., Karasz F.E., MacKnight W.J. (1993). Fourier transform infra-red spectroscopy on the thermo-oxidative degradation of polybenzimidazole and of a polybenzimidazole/polyetherimide blend. Polymer.

[39] Lassettre E.N. (1937). The hydrogen bond and association. Chem. Rev..

[40] Zhuang Y., Gu Y. (2012). Novel poly(benzoxazole-etherimide) copolymer for two-layer flexible copper-clad laminate. J. Macromol. Sci. Part B Phys..

[41] Jou J.H., Huang P.T. (1991). Effect of thermal curing on the structures and properties of aromatic polyimide films. Macromolecules.

[42] Hasegawa M., Matano T., Shindo Y., Sugimura T. (1996). Spontaneous molecular orientation of polyimides induced by thermal imidization. 2. In-plane orientation. Macromolecules.

[43] Hasegawa M., Okuda K., Horimoto M., Shindo Y., Yokota R., Kochi M. (1997). Spontaneous molecular orientation of polyimides induced by thermal imidization. 3. Component chain orientation in binary polyimide blends. Macromolecules.

[44] Tang Y., Yao H., Xu W., Zhu L., Zhang Y., Jiang Z. (2023). Side-chain-type High Dielectric-constant Dipolar Polyimides with Temperature Resistance. Macromol. Rapid Commun..

[45] Tong H., Ahmad A., Fu J., Xu H., Fan T., Hou Y., Xu J. (2019). Revealing the correlation between molecular structure and dielectric properties of carbonyl-containing polyimide dielectrics. J. Appl. Polym. Sci..

[46] Watanabe Y., Shibasaki Y., Ando S., Ueda M. (2005). Synthesis and characterization of polyimides with low dielectric constants from aromatic dianhydrides and aromatic diamine containing phenylene ether unit. Polymer.

[47] Goto K., Kakuta M., Inoue Y., Matsubara M. (2000). Low dielectric and thermal stable polyimides with fluorene structure. J. Photopolym. Sci. Technol..

[48] Goto K., Inoue Y., Matsubara M. (2001). Low dielectric and thermally stable polyimides with fluorene structure (II) relationship between chemical structure and dielectric constant. J. Photopolym. Sci. Technol..

[49] Zhuang Y., Liu X., Gu Y. (2012). Molecular packing and properties of poly(benzoxazole-benzimidazole-imide) copolymers. Polym. Chem..

[50] Yan X., Dai F., Ke Z., Yan K., Chen C., Qian G., Li H. (2022). Synthesis of Colorless Polyimides with High *T*_g_ from Asymmetric Twisted Benzimidazole Diamines. Eur. Polym. J..

[51] Lian M., Lu X., Lu Q. (2018). Synthesis of superheat-resistant polyimides with high *T*_g_ and low coefficient of thermal expansion by introduction of strong intermolecular interaction. Macromolecules.

[52] Ou X., Chen S., Lu X., Lu Q. (2021). Enhancement of Thermal Conductivity and Dimensional Stability of Polyimide/Boron Nitride Films Through Mechanochemistry. Compos.Commun..

[53] Cheng Y., Dong J., Yang C., Wu T., Zhao X., Zhang Q. (2017). Synthesis of poly(benzobisoxazole-co-imide) and fabrication of high-performance fibers. Polymer.

