# Supplementary material for: The Pivotal Role of Benzimidazole in Improving the Thermal and Dielectric Performance of Upilex-Type Polyimide
Source: Polymers (Basel). 2023 May 17;15(10):2343. doi: 10.3390/polym15102343 (PMC10223674; doi:10.3390/polym15102343)
Supplement: Supplementary file 1 [file polymers-15-02343-s001.zip › polymers-2351968-supplementary.pdf]

# Supplementary Materials: The Pivotal Role of Benzimidazole in Improving the Thermal and Dielectric Performance of Upilex-Type Polyimide

**Meng Lian [1], Fei Zhao [1], Jun Liu [1], Faqin Tong [2], Lingbin Meng [1], Yongqi Yang [1] and Feng Zheng [3,*]**

[1] Shandong Engineering Laboratory for Clean Utilization of Chemical Resources, Weifang University of Science and Technology, Weifang 262700, China; menglian@alumni.sjtu.edu.cn (M.L.); zhaofei@wfust.edu.cn (F.Z.); junliu@smail.nju.edu.cn (J.L.); mlb8124@126.com (L.M.); yongqiyang@wfust.edu.cn (Y.Y.)
[2] Shanghai Sinochem Technology Co., Ltd., Kangwei Road 299, Pudong New District, Shanghai 201210, China; lanfeng@tongji.edu.cn
[3] School of Chemical Science and Engineering, Tongji University, Siping Road 1239, Shanghai 200092, China
* Correspondence: fzheng@tongji.edu.cn

## The synthesis and characterization of 4,4'-[5,5'-bi-1H-benzimidazole]-2,2'-diylbis-benzenamine (BB)

The synthesis procedure was shown in Scheme S1 as follows: 160 g Poly(phosphoric acid), 10.7 g 3,3′-diaminobenzidine (50 mmol) and 13.7 g 4-aminobenzoic acid (100 mmol) were added to a dried 500 mL three-neck flask equipped with a thermometer, condenser, and a mechanical stirrer. The mixture was heated to 200 °C and reacted at this temperature for 12 h. Then cooled to 60 °C and poured into ice-cold water with rapid stirring. Finally, the precipitate formed was soaked in a 5% sodium hydrogen carbonate solution overnight. The crude product was purified by chromatography eluting with ethyl acetate/ethanol (3:1, v/v) to afford a light yellow solid. The crude product dissolved DMSO and then precipitated in water to obtain a white solid. $^1$H NMR (500 MHz, DMSO-$d_6$) δ 12.51 (s, 2H), 7.88 (d, $J$ = 8.5 Hz, 4H), 7.73 (s, 2H), 7.57 (d, $J$ = 7.6 Hz, 2H), 7.47 (dd, $J$ = 8.3, 1.5 Hz, 2H), 6.70 (d, $J$ = 8.6 Hz, 4H), 5.63 (s, 4H).

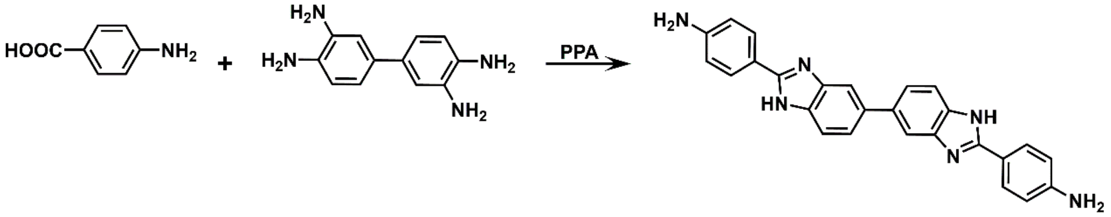


**Scheme S1.** Synthesis of BB.

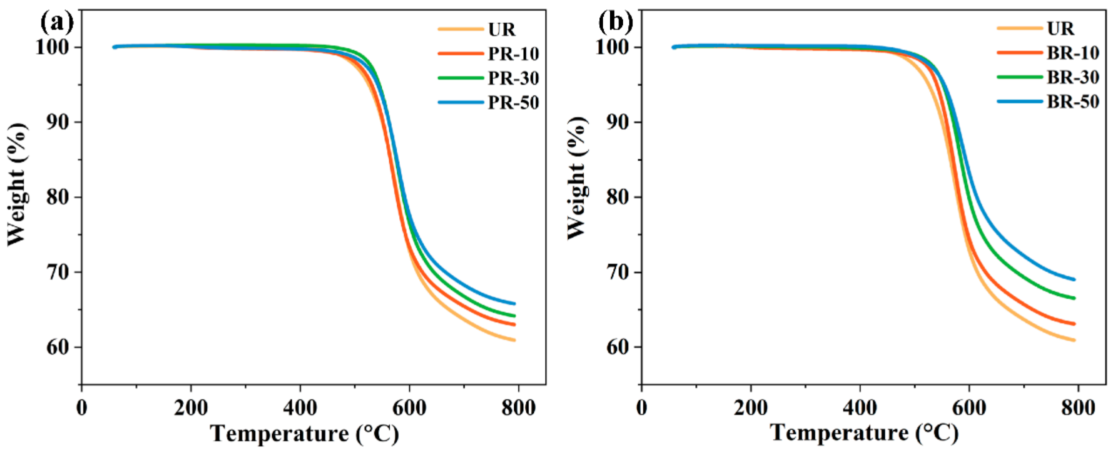


**Figure S1**. TGA curves of PI films (a) series PR and (b) series BR.

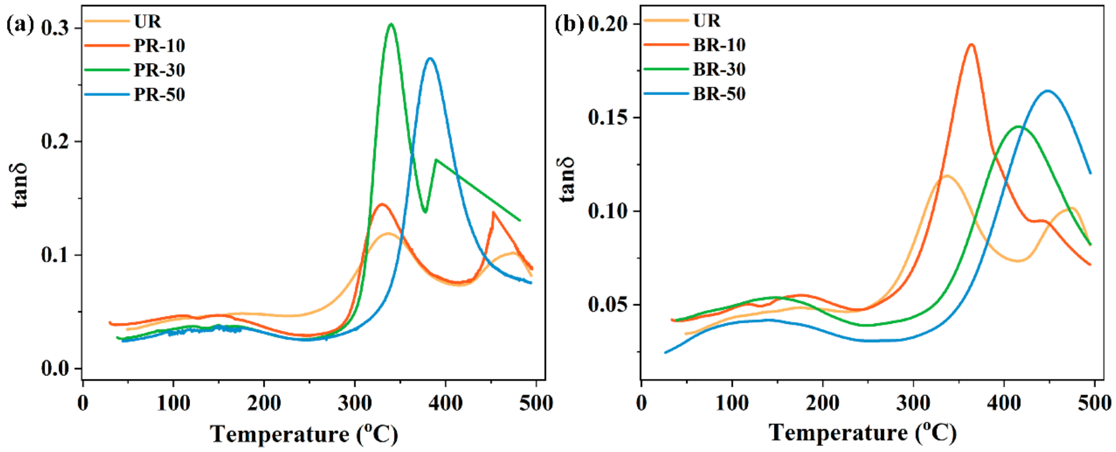


**Figure S2**. DMA curves of PI films(a) series PR and (b) series BR.

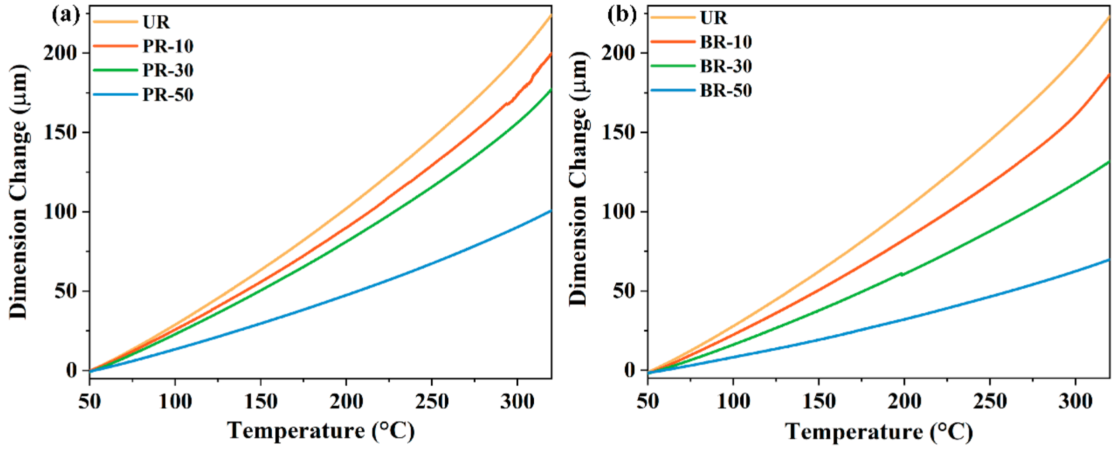


**Figure S3**. TMA curves of PI films(a) series PR and (b) series BR.

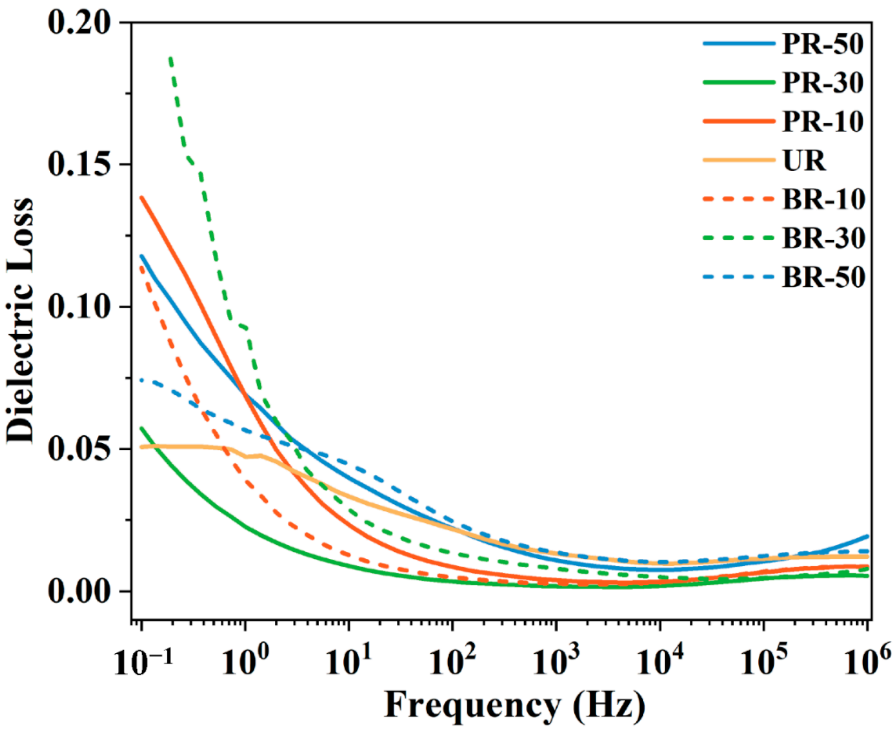


**Figure S4.** Frequency-scan broadband dielectric loss spectroscopy of polyimides.
